# Ecological Niche Modeling of the Narrow-Range Endangered Endemic *Lepidium olgae* in Uzbekistan

**DOI:** 10.3390/plants15071125

**Published:** 2026-04-07

**Authors:** Khusniddin Abulfayzov, Bekhruz Khabibullaev, Khabibullo Shomurodov, Natalya Beshko, Suluv Sullieva, Yaoming Li, Lianlian Fan

**Affiliations:** 1 Laboratory of Conservation and Ecology of Plants, Institute of Botany Academy of Sciences of Republic Uzbekistan, Tashkent 100125, Uzbekistan; husniddin.abulfayzov1@mail.ru (K.A.); bekh.xabibullaev@mail.ru (B.K.); h.shomurodov@mail.ru (K.S.); natalia_beshko@mail.ru (N.B.); 2 Department of Botany, Faculty of Natural Sciences, Termez State University, Termez 190111, Uzbekistan; info@tersu.uz; 3 Xinjiang Institute of Ecology and Geography, Chinese Academy of Sciences, Urumqi 830011, China; lym@ms.xjb.ac.cn; 4 Research Center for Ecology and Environment of Central Asia, Chinese Academy of Sciences, Urumqi 830011, China

**Keywords:** Central Asia, ensemble SDM, microhabitat stability, mountain ecosystem, narrow-range endemic species

## Abstract

Narrow-range endemic plant species are highly sensitive to environmental variability due to their restricted distributions and narrow ecological niches, yet quantitative assessments of such species in Central Asian mountain ecosystem remain limited. This study applied an ensemble species distribution modeling (SDM) approach to assess the ecological constraints and conservation efforts of *Lepidium olgae*, a strict endemic species of the Nuratau Mountains in Uzbekistan. Species occurrence records from field surveys and herbarium data were integrated with remotely sensed climatic, vegetation, topographic, soil, and atmospheric variables. Parsimonious models (Generalized Linear Model (GLM), Maximum Entropy (MaxEnt), Multiple Adaptive Regression Splines (MARS), Surface Range Envelope (SRE)) were implemented in BIOMOD2 4.3.4, and ensemble predictions were used to reduce algorithmic uncertainty and identify core habitat patterns. Results showed that wet-season precipitation was the dominant driver of species distribution, followed by vegetation productivity (NDVI) and thermal stability, indicating a strong dependence on moisture availability and stable microhabitats. Ensemble projections revealed a highly fragmented potential distribution, with suitable habitats covering only 8% of the reserve area, closely matching the observed distribution of 6.5%. This strong spatial overlap confirms a narrowly constrained realized ecological niche. These findings highlight the critical role of microhabitat stability for the persistence of *Lepidium olgae* and provide a spatially explicit basis for prioritizing in situ conservation and guiding model informed translocation efforts.

## 1. Introduction

The conservation of narrow range and endemic plant species is a central challenge in biodiversity science, as such species often represent unique evolutionary lineages with highly constrained ecological niches. Due to their restricted distributions and limited ecological amplitudes, endemic plants are strongly influenced by climatic, topographic, edaphic, and microhabitat conditions, making them particularly sensitive to environmental variability. As a result, understanding the environmental drivers that shape their spatial distributions is essential for both ecological theory and conservation planning [1,2].

Mountain ecosystems are characterized by pronounced environmental heterogeneity, where sharp gradients in temperature, moisture availability, and soil properties occur over short spatial distances. These gradients frequently confine endemic plant species to a limited number of environmentally stable microhabitats, resulting in fragmented distributions and narrow realized niches. Recent studies have shown that both climatic variability and climate change can substantially alter habitat suitability for narrow endemic species in mountainous regions, emphasizing the importance of quantifying species–environment relationships at appropriate spatial scales [3,4]. In parallel, vegetation dynamics within major river basins have been shown to be closely linked to broader environmentally driven ecological degradation, pose increasing challenges for the long-term persistence of narrow-range plant species [5,6].

Central Asian Mountain systems, which harbor a considerable number of narrow-range species adapted to arid and semi-arid environments, remain underrepresented in global studies of endemic plant ecology, where seasonal moisture availability and thermal stability are increasingly recognized as primary constraints on species persistence [7]. Despite this recognition, quantitative, species-specific assessments remain limited [8,9]. This lack of detailed ecological analyses hampers the development of robust, spatially explicit conservation strategies for endemic flora in Central Asia. *Lepidium olgae* Regel (Brassicaceae) is a typical narrow endemic plant species restricted to the Nuratau Mountain Range, characterized by a highly limited distribution and a strong association with specific environmental conditions. The species is a perennial herbaceous plant adapted to arid mountain environments, typically occurring on rocky slopes and shallow soils. The species exhibits morphological and physiological adaptations to drought stress, including reduced leaf surface area and efficient water-use strategies. It is commonly associated with xerophytic plant communities and occupies environmentally stable microhabitats within semi-arid mountain ecosystems. Owing to its restricted range and ecological specialization, the species represents a suitable model for investigating the environmental determinants of endemic plant distributions in Central Asian Mountain ecosystems.

Ecological niche modeling and species distribution modeling (ENM/SDM) provide powerful tools to quantify the environmental constraints governing narrow endemic species. By linking species occurrence records with climatic, topographic, soil, and microhabitat variables, SDMs enable the identification of key ecological drivers, the delineation of suitable habitats, and the assessment of spatial overlap between realized and potential distributions [2,10,11,12,13]. Ensemble modeling frameworks, such as BIOMOD2, integrate multiple algorithms to reduce individual model bias and improve the robustness of predictions [14,15].

Against this background, the objectives of the present study are to (i) model the potential distribution of *Lepidium olgae* using an ensemble SDM approach, (ii) quantify the relative importance of climatic, topographic, and microhabitat variables shaping its ecological niche, and (iii) assess the spatial correspondence between observed and predicted distributions. By focusing on a narrow endemic species in a data-deficient region, this study addresses a critical knowledge gap in the ecology of Central Asian mountain endemics and provides a scientifically grounded basis for spatially explicit conservation planning.

Accordingly, we hypothesize that the distribution of *Lepidium olgae* is primarily controlled by seasonal moisture availability and microhabitat stability, with vegetation cover (NDVI) and temperature variability acting as key limiting factors.

## 2. Results

### 2.1. Model Performance

Model performance was evaluated using repeated random split cross-validation, with 80% of the data used for model calibration and 20% for validation, repeated five times. Performance metrics were calculated exclusively on validation data to assess the generalization ability of individual models.

Across the four modeling algorithms, validation AUC values indicated moderate discriminatory ability, ranging from 0.56 ± 0.09 for the Surface Range Envelope (SRE) model to 0.79 ± 0.11 and 0.79 ± 0.15 for the MaxEnt and MARS models, respectively. The Generalized Linear Model (GLM) showed slightly lower but still acceptable performance (AUC = 0.71 ± 0.13).

Mean TSS values ranged from 0.12 ± 0.17 for SRE to 0.41 ± 0.36 for MARS, while GLM and MaxEnt showed intermediate values (0.38 ± 0.27 and 0.22 ± 0.27, respectively). The relatively low TSS values observed for SRE reflect its conservative envelope-based algorithm, which delineates suitable environmental ranges without optimizing probabilistic discrimination, rather than indicating predictive instability. Kappa values were generally low to moderate, varying between 0.15 ± 0.31 for MaxEnt and 0.38 ± 0.27 for MARS, further reflecting uncertainty associated with small sample sizes and the use of random pseudo-absence data.

**Figure 1:**
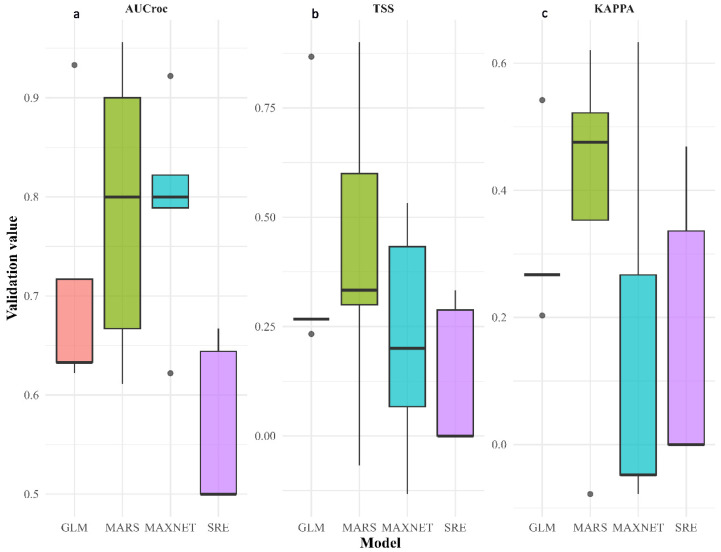
Cross-validated performance of individual ecological niche models. Model accuracy was assessed using (**a**) AUC, (**b**) TSS, and (**c**) Kappa metrics across multiple cross-validation runs for GLM, MARS, MaxEnt, and SRE algorithms. Boxes represent the interquartile range, horizontal lines indicate median values, whiskers show the range, and dots represent outliers. Overall, MaxEnt and MARS models demonstrated higher predictive performance compared to GLM and SRE.

Overall, performance metrics showed substantial variability across cross-validation runs, particularly for threshold-dependent indices, highlighting the inherent uncertainty in modeling narrow endemic species with limited occurrence records (Figure 1). Therefore, individual model performance was interpreted cautiously, and subsequent analyses focused on spatial agreement among models rather than on identifying a single “best” model or claiming high predictive accuracy. Modest model performance reflects the ecological rarity and restricted niche breadth of the species rather than methodological failure, a common pattern in SDM studies of narrow-range endemic species.

### 2.2. Environmental Drivers and Ecological Responses of Lepidium olgae Distribution

**Figure 2:**
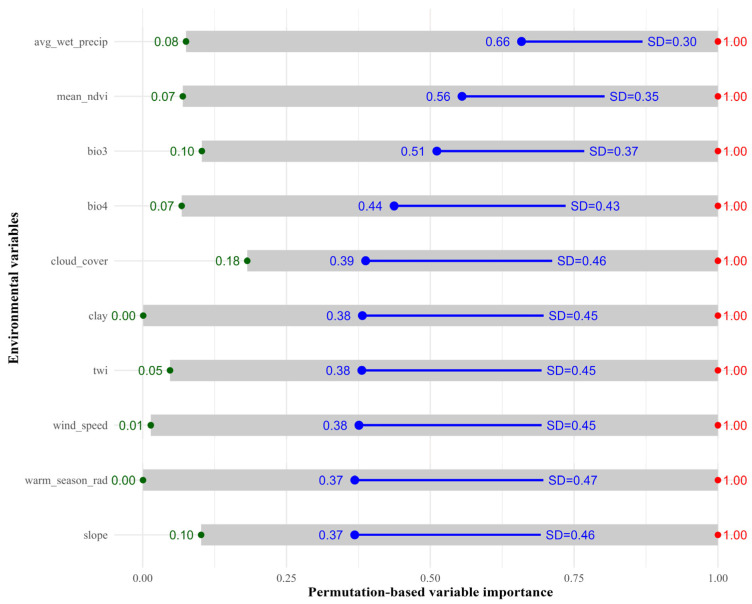
Permutation-based variable importance for *Lepidium olgae* across environmental predictors.

Permutation-based variable importance analysis was applied to quantify the relative contribution of environmental predictors to the spatial distribution of *Lepidium olgae*. Predictor importance values were calculated through random permutation and averaged across four modeling algorithms and five cross-validation runs (RUN1–RUN5), allowing simultaneous evaluation of the magnitude and stability of effects (Figure 2). Among all predictors, avg_wet_precip exhibited the highest mean importance (mean = 0.66, SD = 0.30; min = 0.08; max = 1.00), identifying wet-season precipitation as the dominant environmental constraint on the species’ distribution. The relatively high variability among runs suggests spatial heterogeneity in moisture limitation and context-dependent control by water availability. mean_ndvi ranked second in importance (mean = 0.56, SD = 0.35; min = 0.07; max = 1.00), highlighting the strong association between species occurrence and vegetation productivity. Climatic stability further influenced distribution patterns: bio3 (isothermality) showed consistently high importance (mean = 0.51, SD = 0.37), whereas bio4 (temperature seasonality) displayed slightly lower but highly variable importance (mean = 0.44, SD = 0.43), indicating the sensitivity of *Lepidium olgae* to pronounced thermal fluctuations. Other predictors, including cloud_cover, clay, twi, wind_speed, warm_season_rad, and slope, exhibited moderate mean importance values with wide ranges, suggesting secondary, context-dependent effects.

Ecological response curves derived from GLM, MaxEnt, MARS, and SRE models provided further insight into the functional relationships between environmental gradients and habitat suitability. For avg_wet_precip, GLM, MaxEnt, and MARS models consistently indicated a positive response: predicted presence probability remained low (≈0.15–0.25) at lower precipitation levels (≈1.08–1.10) but increased steadily with higher wet-season precipitation, reaching ≈0.45–0.60. This pattern confirms moisture availability during the wet season as a key limiting factor for *Lepidium olgae*. The nearly flat response observed in the SRE model reflects its envelope-based algorithm, which does not estimate probabilistic gradients. Increasing bio3 (isothermality) was associated with higher predicted suitability in GLM and MaxEnt models, while the MARS model captured this relationship in a threshold-like manner. In contrast, bio4 (temperature seasonality) exerted a strong negative effect across all probabilistic models, with predicted suitability declining sharply from −0.40–0.50 under low seasonality to −0.10–0.20 at higher seasonality values, indicating intolerance to strong thermal variability.

**Figure 3:**
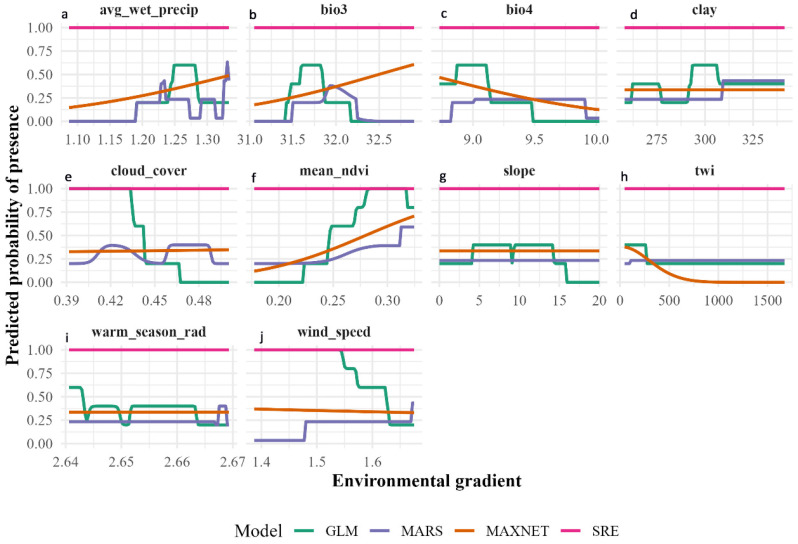
Response curves of *Lepidium olgae* showing the relationship between predicted probability of presence and environmental gradients derived from individual niche models (GLM, MARS, MaxEnt, and SRE) for (**a**) avg_wet_precip, (**b**) bio3, (**c**) bio4, (**d**) clay, (**e**) cloud_cover, (**f**) mean_ndvi, (**g**) slope, (**h**) twi, (**i**) warm_season_rad, and (**j**) wind_speed.

Vegetation-related effects were also pronounced. mean_ndvi emerged as one of the strongest positive predictors, with predicted presence probability increasing to −0.70–0.80 in some models, underscoring the close association of *Lepidium olgae* with well-developed vegetation cover and stable microclimatic conditions. In contrast, twi showed a consistent negative response across all models, suggesting a preference for well-drained habitats rather than persistently water-accumulating areas. Responses to slope and clay content were weak, while cloud_cover, warm_season_rad, and wind_speed exhibited low-amplitude effects, indicating that these factors play secondary roles in shaping the species’ distribution. Overall, the strong consistency in response direction among GLM, MaxEnt, and MARS models supports the biological robustness of the inferred ecological relationships and confirms that moisture availability, vegetation cover, and climatic stability collectively define the core environmental niche of *Lepidium olgae* (Figure 3).

### 2.3. Ensemble Prediction, Model Agreement, and Spatial Uncertainty of Lepidium olgae

**Figure 4:**
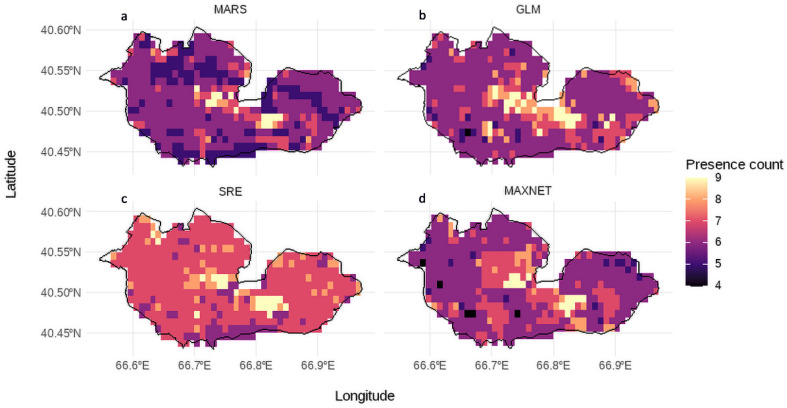
Spatial agreement among individual niche models, including (**a**) MARS, (**b**) GLM, (**c**) SRE, and (**d**) MaxEnt, showing the number of models predicting suitable habitat for *Lepidium olgae* within the study area.

The potential distribution of *Lepidium olgae* was assessed using an ensemble modeling framework that integrated the outputs of the MARS, GLM, SRE, and MaxEnt algorithms through an equal-weight ensemble mean (EMmean), thereby reducing algorithm-specific uncertainty and providing a more robust representation of habitat suitability across the study area. Model agreement analysis revealed moderate to high spatial consistency among individual algorithms, with most of the study area exhibiting agreement levels of 6–8 models predicting suitable conditions (Figure 4). The highest agreement zones, where nearly all models consistently identified suitable habitat, represent ecologically stable niches, whereas areas with lower agreement (4–5 models) correspond to environmentally marginal or uncertain conditions.

Distinct differences among algorithms were observed, with MARS producing more conservative and fragmented suitability patterns, GLM predicting broader areas with high consensus, SRE showing widespread suitability due to its generalized nature, and MaxEnt generating well-defined and spatially clustered predictions in ecologically optimal environments. Overall, zones of high model agreement were primarily associated with optimal elevation, temperature, and moisture conditions and lower levels of anthropogenic pressure, indicating the most reliable and stable habitats for *Lepidium olgae*, while low-agreement areas are best interpreted as transitional zones characterized by higher ecological uncertainty.

**Figure 5:**
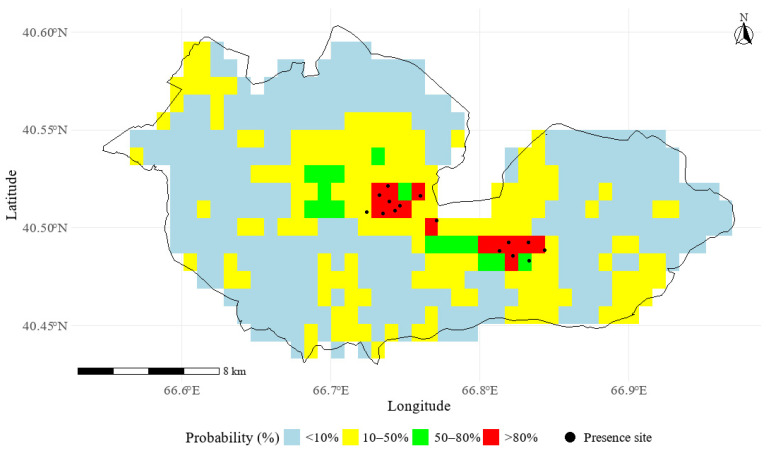
Distribution of ensemble-predicted presence probability of *Lepidium olgae*.

The ensemble probability map further indicated that 61.2% of the study area exhibited a predicted occurrence probability below 10%, reflecting very low habitat suitability across large portions of the landscape. Moderately suitable habitats (10–50%) accounted for the largest proportion of the study area (33%), representing areas where species occurrence may depend on local environmental filters such as soil properties, moisture availability, and microhabitat conditions (Figure 5). In contrast, regions with high (50–80%) and very high (>80%) predicted suitability were spatially restricted, covering only 3.2% and 2.6% of the study area, respectively, and occurring as small, fragmented patches where optimal ecological conditions converge. The proportional area and spatial extent of each probability class are summarized in Table 1. The limited extent of these high-suitability areas confirms that *Lepidium olgae* occupies a narrow ecological niche within the region.

**Figure 6:**
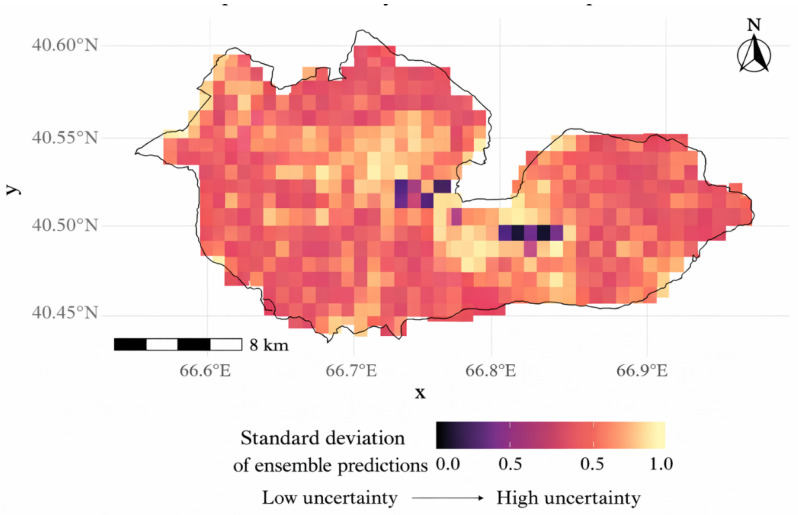
Spatial uncertainty of the ensemble SDM prediction for *Lepidium olgae*. Uncertainty represents inter-model variability across modeling algorithms and cross-validation runs, with higher values indicating lower prediction confidence.

Spatial uncertainty analysis revealed heterogeneous confidence in ensemble predictions across the study area. Areas with high predicted habitat suitability generally exhibited low uncertainty, particularly within the core distribution range of *Lepidium olgae*, whereas peripheral and moderately suitable areas showed higher uncertainty, reflecting increased disagreement among individual models and cross-validation runs (Figure 6). The spatial correspondence between observed occurrences and ensemble-predicted suitable areas further supports the reliability of the modeling framework. This pattern underscores the importance of cautious interpretation in transitional zones, while highlighting high-suitability, low-uncertainty areas as priority targets for conservation planning and further ecological investigation.

### 2.4. Spatial Correspondence Between Observed and Predicted Distributions

According to the ensemble modeling results, the potential distribution area of *Lepidium olgae* within the Nurota Nature Reserve was estimated at 30.29 km^2^, accounting for 8% of the total reserve area. This potential distribution was derived from the ensemble-based habitat suitability map generated using parsimonious ecological niche models and subsequently converted into a binary suitability map using a TSS-based threshold.

The observed (actual) distribution of *Lepidium olgae* was determined based on georeferenced field survey data collected along systematic transects and supplemented with verified herbarium records. These occurrence data were spatially summarized and represented as polygons in a GIS environment to delineate the currently documented distribution of the species. As a result, the observed distribution covered 25.04 km^2^, representing 6.5% of the Nurota region (Table 2).

**Figure 7:**
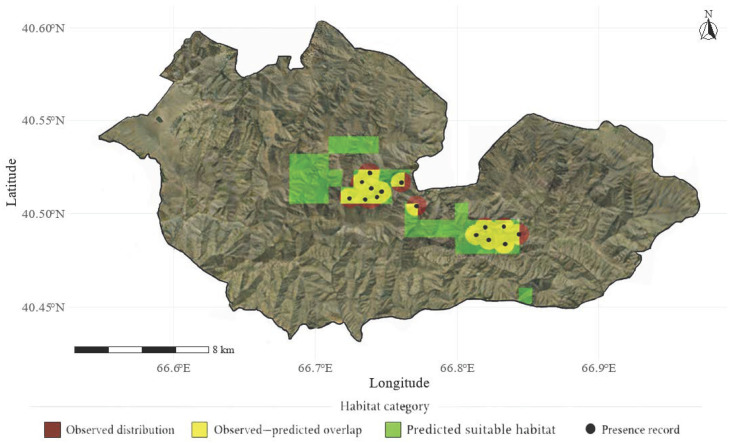
Spatial distribution of *Lepidium olgae* in the Nurota Nature Reserve.

Although the potential and observed distribution areas did not fully coincide, a substantial spatial overlap was detected between them. The overlapping area amounted to 24.69 km^2^, corresponding to 81.5% of the predicted potential distribution and 97.2% of the observed distribution. This high degree of spatial correspondence indicates that the ensemble model successfully captured the core environmental conditions associated with the current distribution of *Lepidium olgae* (Figure 7). Ecologically, the strong overlap between predicted and observed distributions suggests that the environmental gradients included in the model—primarily climatic, vegetation-related, and topographic variables—play a major role in shaping the spatial pattern of *Lepidium olgae* within the study area. Nevertheless, areas where the species was observed but not predicted by the model imply that additional factors not explicitly represented in the modeling framework, such as fine-scale soil heterogeneity, microtopography, or local biotic interactions, may also influence species occurrence.

Conversely, regions predicted as suitable but currently lacking confirmed records may represent environmentally favorable habitats that remain unoccupied due to dispersal limitations, historical factors, or unmeasured ecological constraints. Together, these results highlight both the strengths and limitations of the ensemble modeling approach and emphasize the importance of integrating field-based observations with spatial modeling to better understand the distribution patterns of narrow endemic species such as *Lepidium olgae*.

## 3. Discussion

### 3.1. Main Ecological Drivers Shaping the Distribution of Lepidium olgae: Comparison with Existing Literature

The results of this study indicate that the spatial distribution of *Lepidium olgae* is primarily influenced by moisture availability, vegetation-related conditions, and thermal stability. These findings demonstrate a high degree of consistency with high-impact studies addressing ecological constraints of narrow endemic plant species in mountainous environments. Among the environmental predictors, wet-season precipitation, NDVI, and temperature seasonality were identified as the most influential variables, highlighting the critical role of water availability and microclimatic stability in shaping the ecological niche of *Lepidium olgae*. In addition, long-term meteorological data (1981–2020) from the Nurata and Yangiqishloq stations indicate a consistent warming trend accompanied by a decline in precipitation in the study region. Mean annual temperature increased by +0.006 °C yr^−1^ at Yangiqishloq (total +0.24 °C) and by +0.022 °C yr^−1^ at Nurata (total +0.9 °C), with a higher frequency of warm years after the early 2000s.

Precipitation exhibited strong interannual variability but showed an overall decreasing trend. At Yangiqishloq, annual precipitation declined by −0.059 mm yr^−1^ (total −2.4 mm), whereas a more pronounced decrease was observed at Nurata (−0.608 mm yr^−1^, total −24 mm).

These climatic trends reinforce the importance of moisture availability and thermal stability identified in this study, as increasing temperatures and declining precipitation may intensify water limitation and further constrain the ecological niche and persistence of *Lepidium olgae*. The identification of wet-season precipitation (avg_wet_precip) as the most influential predictor is in strong agreement with previous research conducted in semi-arid and continental mountain systems. In alpine and high-mountain ecosystems, plant distribution is often more strongly constrained by growing season water availability than by total annual precipitation [16,17]. Similarly, studies from semi-arid mountain regions have shown that seasonal drought can lead to population fragmentation and restrict species persistence [18,19].

In the present study, the high relative importance of avg_wet_precip indicates that *Lepidium olgae* is highly sensitive to water limitation during the vegetative period and that its ecological niche is tightly constrained by seasonal moisture availability. This pattern is consistent with the concept of a “water-limited niche,” which has been widely documented for narrow-range species inhabiting climatically stressful environments [2,20].

Vegetation-related variables, particularly NDVI, have frequently been interpreted as integrative indicators of microhabitat quality and microclimatic stability [21,22]. Studies conducted in mountainous and arid regions demonstrate that areas with higher NDVI values provide greater buffering against thermal extremes and improved soil moisture retention [7]. The results obtained for *Lepidium olgae* align with this mechanism, indicating a clear preference for vegetation protected microhabitats over open, environmentally stressful surfaces. This indicates that vegetation density (NDVI) plays a crucial role in providing suitable microhabitats for *Lepidium olgae*, supporting its survival under harsh semi-arid conditions.

Temperature-related variables further supports this interpretation. Previous studies on mountain endemics emphasize the importance of high isothermality (bio3) and low temperature seasonality (bio4) for maintaining ecological stability [23,24]. In this study, the consistently negative effect of temperature seasonality suggests that *Lepidium olgae* is poorly adapted to pronounced thermal fluctuations. Such sensitivity is characteristic of relict and narrow endemic mountain species whose persistence depends on Temperature related microclimatic conditions [25].

Overall, these results quantitatively demonstrate the importance of seasonal moisture availability and microhabitat stability for *Lepidium olgae*, thereby contributing new evidence to the limited body of knowledge on endemic plant species in Central Asian Mountain ecosystems.

### 3.2. Ecological Interpretation Based on Response Curves

Response curves provide mechanistic insight into the ecological behavior of *Lepidium olgae* along key environmental gradients. Across GLM, MaxEnt, and MARS models, habitat suitability consistently increased with rising wet-season precipitation, a pattern consistent with findings from other mountain regions hosting narrow endemic plant species [19,26].

The sharp decline in predicted suitability at low avg_wet_precip values identifies these conditions as ecological stress zones for *Lepidium olgae*. This response underscores the dominant role of water limitation as a primary ecological constraint on species distribution.

Similarly, NDVI exhibited a strong positive relationship with habitat suitability. High NDVI values, which are commonly associated with favorable microclimatic conditions and enhanced soil moisture availability, corresponded to markedly increased occurrence probabilities [21,22]. These results suggest that optimal habitat conditions for *Lepidium olgae* are found in vegetation-buffered microhabitats that mitigate environmental stress.

In contrast, temperature seasonality had a strong negative influence across all probabilistic models. This response pattern aligns with the concept of “thermal stability specialization,” commonly reported for narrow endemic and relict species in mountainous regions [24,27]. Collectively, these findings indicate that *Lepidium olgae* is particularly sensitive to climatic variability and may be vulnerable to future climate change.

### 3.3. Study Limitations

The observed spatial uncertainty patterns are consistent with the limited number of occurrence records and the ecological specialization of *Lepidium olgae*, which increases model sensitivity model sensitivity under marginal environmental conditions. Several limitations should be acknowledged. First, as noted in numerous studies on rare and endemic species, limited occurrence records can constrain model performance and increase uncertainty, particularly for threshold-dependent metrics [28]. The observed variability in evaluation metrics in this study is consistent with the limited number of occurrence records available. Second, the spatial resolution of global and regional environmental layers may be insufficient to capture fine-scale microhabitat heterogeneity, including subtle topographic or edaphic variations [2]. Consequently, some ecologically suitable micro-sites may remain undetected. In addition, as the study area represents the northernmost part of the Nuratau Mountain range, it may be influenced by specific geographic and climatic conditions. This region may act as a natural ecological boundary, limiting species dispersal and contributing to the fragmented distribution pattern observed in Figure 7.

Furthermore, the use of a grid-based approach and the spatial resolution of environmental data may introduce a smoothing effect, potentially reducing the representation of fine-scale environmental heterogeneity. While explicit grid smoothing was not applied in this study, future analyses could explore smoothing techniques to better capture spatial continuity in habitat suitability patterns. As with most modeling studies on rare endemic species, limited occurrence data constrain predictive accuracy; therefore, model outputs should be interpreted as relative habitat suitability patterns rather than precise predictions of presence.

Finally, Uncertainty associated with algorithm selection and pseudo-absence generation is well documented in species distribution modeling [29]. To mitigate these effects, an ensemble modeling approach was adopted, as recommended by previous high-impact studies, which results in more robust and stable spatial predictions [20,30].

### 3.4. Conservation Implications

Areas of high suitability that are associated with elevated uncertainty should be interpreted cautiously and prioritized for targeted field validation rather than immediate conservation action. The ensemble-based habitat suitability maps produced in this study should be regarded as screening level tools rather than definitive predictors of species presence [31]. While they provide valuable spatial guidance for conservation planning, such maps cannot replace detailed, field based assessments. Higher habitat suitability was concentrated in the central part of the Nuratau Mountains, where relatively higher moisture and topographic heterogeneity create favorable microhabitats [7].

Areas identified as highly suitable yet lacking confirmed records may represent underexplored or cryptic populations, as shown in similar ecological niche modeling studies [32]. Therefore, these zones should be prioritized for future field validation and long-term monitoring. Such areas may function as critical ecological cores for the long-term persistence of *Lepidium olgae* and therefore deserve particular attention in conservation planning. Integrating ensemble based predictions with uncertainty aware interpretation provides a pragmatic and scientifically robust framework for guiding conservation actions for narrow endemic species under data limited conditions. Although maps of individual environmental variables were not included, future studies may benefit from providing spatial visualizations of key predictors to further support ecological interpretation.

## 4. Materials and Methods

### 4.1. Study Area

**Figure 8:**
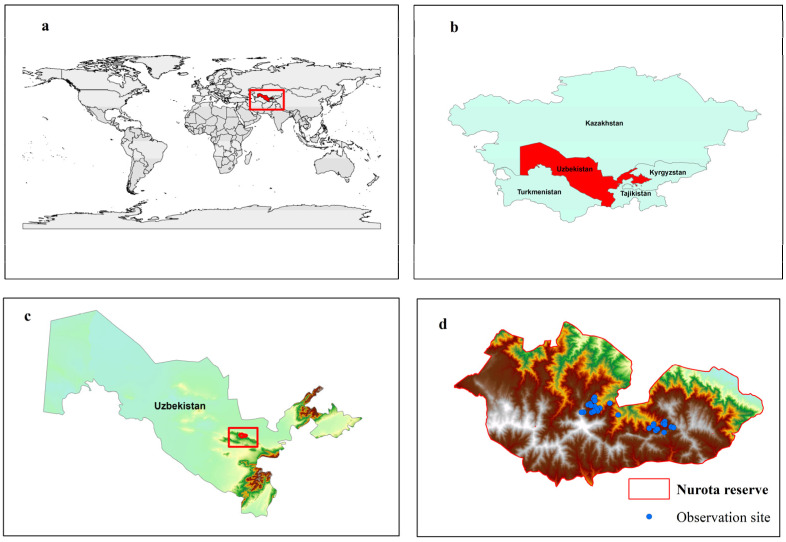
Location of the study area showing Uzbekistan in the global (**a**) and Central Asia (**b**) context, the study area within Uzbekistan (**c**), and the Nurota Nature Reserve with observation sites ((**d**), UTM Zone 41N). Colors represent elevation gradients, with darker shades indicating higher elevations. The red rectangle indicates the location of the study area, and blue points represent observation sites.

The study area is located in Uzbekistan, Central Asia, which places it within the broader arid and semi-arid mountain systems of the region. The Nuratau State Nature Reserve is in central Uzbekistan and represents a key center of biodiversity and endemism in Central Asia [33,34]. Established in 1975, the reserve covers 17,752 ha and is characterized by complex topography and strong altitudinal gradients that generate pronounced local microclimatic heterogeneity. The flora includes approximately 835 vascular plant species, of which 33 are listed in the Red Data Book of the Republic of Uzbekistan [33,35,36]. *Lepidium olgae* is strictly confined to the Nuratau State Nature Reserve and has not been recorded outside its boundaries, making this area the exclusive focus of the present study (Figure 8).

The climate is sharply continental and spatially heterogeneous. Mean annual air temperature in the lower mountain belt ranges from 15 to 20 °C, with average January temperatures of 1–8 °C and mean July temperatures of approximately 29 °C. Extreme temperatures range from −32 °C to +43 °C [33].

Long-term meteorological data (1981–2020) from the Nurata and Yangiqishloq stations were obtained from the Uzbekistan Hydrometeorological Service (Uzhydromet) and used to characterize regional climatic conditions. The combined effects of rising temperatures and decreasing, increasingly variable precipitation indicate intensifying aridization processes in the Nuratau region, with direct implications for habitat suitability of narrow-range and drought-sensitive plant species such as *Lepidium olgae.* Meteorological station data were used solely to characterize local climatic trends and were not employed as predictors in species distribution modeling due to their limited spatial coverage.

### 4.2. Study Species

**Figure 9:**
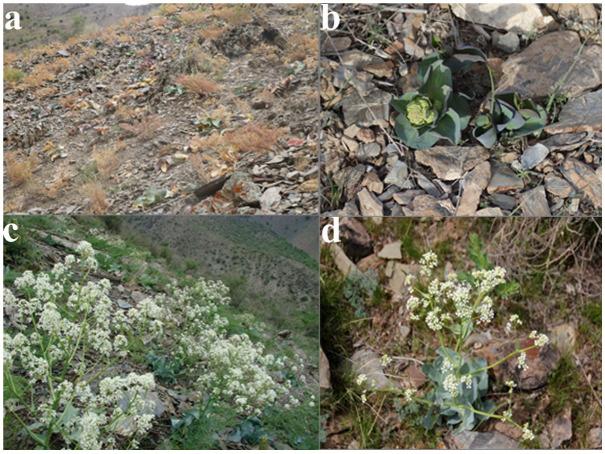
*Lepidium olgae* in the Nurota Nature Reserve. Photographs by V. Beshko (**b**–**d**) and H. Abulfayzov (**a**). (**a**) Typical rocky and dry mountain slope habitat where *Lepidium olgae* occurs. (**b**) Individual at the vegetative rosette stage. (**c**) General view of the population during the flowering period. (**d**) Flowering individual showing a developed generative stem and inflorescence.

*Lepidium olgae* Regel (syn. *Stubendorffia olgae*) is a perennial species of the family Brassicaceae and a narrow-ranged endemic species restricted to the Nuratau Mountain Range, Uzbekistan. This species typically occurs on foothill slopes at elevations of 1000–1400 m, preferentially occupying partially saline substrates under arid to semi-arid climatic conditions. *Lepidium olgae* is primarily associated with semi-arid mountain steppe vegetation, characterized by sparse shrub–grass communities, shallow soils, and pronounced environmental heterogeneity. These habitats are typically dominated by xerophytic plant species adapted to water-limited conditions and high temperature variability. It exhibits morphological and physiological adaptations to short-term moisture availability during the growing season and shows high tolerance to elevated temperatures and drought stress [37]. The species flowers from April to May and fruits from May to June. Despite these adaptive traits, *Lepidium olgae* is characterized by a highly restricted distribution range and limited ecological amplitude, which substantially constrain its resilience to environmental variability (Figure 9). *Lepidium olgae* likely follows the C3 photosynthetic pathway, which is typical for species of the Brassicaceae family and for plants inhabiting temperate and semi-arid environments [17]. As a C3 species, it is generally adapted to moderate temperature conditions but may be sensitive to extreme heat and prolonged drought stress. The species shows a clear ecological preference for habitats with seasonal moisture availability, relying on precipitation during the vegetative period. Its occurrence in semi-arid mountain environments suggests adaptation to water-limited conditions, where microhabitat stability and soil moisture retention are likely to play a critical role in its survival. According to the IUCN Red List Categories and Criteria, *Lepidium olgae* is classified as Endangered (EN) under criteria B2ab (ii, iii, iv, v) and C2a (i). The Extent of Occurrence (EOO) is estimated at approximately 12 km^2^, the Area of Occupancy (AOO) at 16 km^2^, and the total population size is estimated at 1000–1500 individuals [34]. The species is included in the Red Data Book of Uzbekistan under second-level protection status, and conservation actions, including population reinforcement and translocation, have been recommended.

### 4.3. Species Occurrence Data

Occurrence records of *Lepidium olgae* were compiled from two complementary sources. A total of 27 occurrence records were used for this study. Of these, 12 were obtained during targeted field surveys conducted by the authors in 2025 within the Nuratau Nature Reserve, and the remaining 15 records were derived from historical herbarium collections, including the National Herbarium of Uzbekistan (TASH) and the Plantarium database. All herbarium-based records were carefully screened for taxonomic reliability and spatial accuracy, and records with uncertain locality information or low positional precision were excluded prior to analysis. To reduce spatial autocorrelation and ensure independence among occurrences, spatial thinning was applied using a minimum distance of 1 km between records, resulting in 15 independent presence points retained for subsequent modeling.

Pseudo absence points were generated using a random sampling approach within the study area while excluding grid cells containing presence records; full details of the pseudo absence strategy and model calibration procedures are provided in the ecological niche modeling section.

Environmental predictors representing climatic, soil, and topographic conditions were selected based on ecological relevance and low intercorrelation, as described in the corresponding section. All data processing and statistical analyses were conducted in the R environment. Given the extremely limited number of occurrence records typical of narrow endemic species [28,31], all subsequent analyses followed a deliberately conservative and parsimonious modeling strategy.

### 4.4. Remote Sensing and Derivation of Environmental Variables

Environmental variables representing topographic, climatic, vegetation, radiation, atmospheric, and soil conditions were compiled to model the potential distribution of *Lepidium olgae.* Data were obtained from multiple global remote-sensing and gridded datasets. Selected variables were accessed and preprocessed using the Google Earth Engine (GEE) platform, while others were downloaded from publicly available data repositories and processed locally. All predictors were harmonized to a common spatial resolution of 1 km [38].

Topographic variables were derived from the Shuttle Radar Topography Mission (SRTM, 30 m) digital elevation model, including elevation, slope, aspect, and the topographic wetness index (TWI) [39]. Vegetation conditions were characterized using MODIS MOD13A2 NDVI products (1 km, 16-day composites) for the growing season (April–September) during 2020–2024, from which maximum, minimum, mean, and range NDVI values were calculated [40].

Radiation variables were derived from NASA GLDAS data (SWdown_f_tavg), including mean annual radiation, maximum radiation, warm-season radiation, and wet-season radiation. Annual radiation was additionally used as a proxy for sunshine duration [41]. Precipitation was represented by mean wet-season precipitation (November–March) calculated from CHIRPS daily data (5 km) for 2020–2024 [42].

Temperature and atmospheric conditions were characterized using mean nighttime land surface temperature from MODIS MOD11A2 (1 km, 8-day composites) [43], wind speed at 10 m height from ERA5 reanalysis data [44], and mean cloud cover derived from the MOD09GA quality assurance (QA) layer [45]. Soil properties, including clay, sand, silt, pH, and organic carbon content at 0–5 cm depth, were obtained from the SoilGrids database at 250 m resolution and subsequently resampled to 1 km to ensure consistency with other predictors [46].

In addition, 19 bioclimatic variables (bio1-bio19) were obtained from WorldClim v2.1 (1 km resolution, 1970–2000) to represent long-term climatic conditions shaping the species’ fundamental climatic niche [47]. These variables are listed in Table S1 (Supplementary Materials). Bioclimatic variables were used to characterize long-term climatic constraints, whereas recent remote-sensing datasets (2020–2024) capture contemporary environmental variability relevant to current habitat conditions. The combination of long-term climatic baselines and contemporary environmental predictors follows standard practice in species distribution modeling, particularly for narrow-range species.

**Figure 10:**
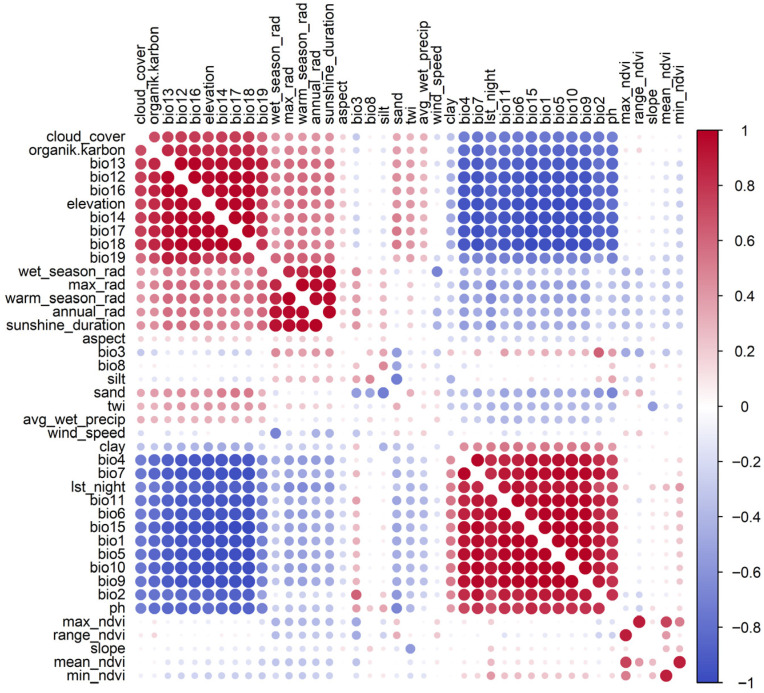
Correlation matrix of environmental predictors. Colors indicate the direction of correlation (red = positive, blue = negative), while circle size represents the strength of correlation, with larger circles indicating stronger relationships.

To reduce multicollinearity, Pearson correlation analysis (Figure 10) (|r| ≥ 0.7) and Variance Inflation Factor (VIF ≤ 5) filtering were applied [48,49], resulting in 15 relatively independent variables. From this reduced set, a parsimonious subset of ten predictors was selected based on ecological relevance, interpretability, and consistency across modeling algorithms, while avoiding over-parameterization given the limited number of occurrence records. The final predictor set included vegetation productivity (mean NDVI); climatic variability and energy availability (BIO3, BIO4, avg_wet_precip, warm-season radiation, and wind speed); soil texture (clay); topographic and hydrological conditions (slope and TWI); and atmospheric modulation (cloud cover).

### 4.5. Ecological Niche Modeling

#### 4.5.1. Biodiversity Modeling Construction

In this study, the potential distribution of *Lepidium olgae* was modeled using the BIOMOD2 framework implemented in R software (R version 4.5.1; biomod2 package version 4.3.4) [14]. Although BIOMOD2 provides a wide range of ecological niche modeling algorithms, only parsimonious methods known to perform robustly with small sample sizes were retained for the final analyses. Accordingly, four algorithms were selected: the Generalized Linear Model (GLM), Maximum Entropy (MaxEnt implementation), Multiple Adaptive Regression Splines (MARS), and the Surface Range Envelope (SRE).

Species occurrence data (presence-only) were combined with randomly generated pseudo-absence data using a 1:10 presence-to-absence ratio. After testing for multicollinearity and ecological relevance, a final set of 10 weakly correlated and biologically meaningful environmental variables was retained for model calibration.

Model evaluation was conducted using repeated random split cross-validation implemented in BIOMOD2, with 80% of the data used for model calibration and 20% for validation, repeated five times. Model performance was assessed exclusively on validation data using the Area Under the Receiver Operating Characteristic Curve (AUC), the True Skill Statistic (TSS), and the Kappa statistic. Full models were not refitted to avoid optimistic bias, ensuring that all performance metrics reflected generalization ability rather than calibration fit.

Ensemble predictions were generated based on spatial consensus among the four individual models using BIOMOD2’s ensemble forecasting procedure. These ensemble maps were interpreted to highlight relative habitat suitability patterns rather than to emphasize absolute predictive accuracy. Ensemble predictions were generated using an equal-weight (EMmean) consensus approach based on continuous suitability outputs. Individual cross-validation runs with TSS < 0.2 were excluded from ensemble construction to minimize the influence of poorly performing partitions; however, the SRE algorithm was retained due to its ecological interpretability as an envelope-based model. AA binary suitability map was subsequently derived from the ensemble predictions using a mean TSS-based (Youden) threshold. To reduce overfitting risk associated with small sample sizes, model complexity was minimized through strict variable filtering, the use of parsimonious algorithms, and an ensemble consensus approach rather than reliance on single-model outputs. Spatial uncertainty was quantified as inter-model variability across modeling algorithms and cross-validation runs and visualized as a spatial uncertainty map.

#### 4.5.2. Spatial Comparison Between Observed and Predicted Distributions

The observed distribution of *Lepidium olgae* was derived from georeferenced occurrence records obtained from systematic field surveys and complemented by verified herbarium data, representing the currently documented occurrences of the species within the study area.

To delineate the spatial extent of the observed distribution, a minimum convex polygon (convex hull) encompassing all confirmed occurrence points was constructed. This polygon represents the spatial envelope supported by empirical observations and does not imply the complete realized distribution of the species.

The predicted potential distribution was represented by the ensemble-based habitat suitability map generated through ecological niche modeling. For visualization and spatial comparison purposes, the continuous ensemble suitability output was converted into a binary map using a mean TSS based (Youden index) threshold (cutoff = 0.432).

A spatial overlay analysis was subsequently performed between the observed distribution polygon and the binary ensemble suitability map to identify areas of spatial agreement and mismatch between documented occurrences and predicted suitable habitats. This analysis was intended to provide a descriptive assessment of spatial correspondence and was not designed to infer causal relationships or to attribute observed patterns to specific environmental or anthropogenic drivers.

## 5. Conclusions

This study provides a comprehensive assessment of the ecological constraints and conservation vulnerability of the narrow endemic plant *Lepidium olgae* in the Nurota Mountain Range using an ensemble species distribution modeling (SDM) framework. The results show that the species occupies an extremely narrow ecological niche, exhibits a highly fragmented distribution, and is strongly dependent on specific environmental conditions. Ensemble models indicate that the observed distribution covers only 6.5% of the reserve area, while potentially suitable habitats account for 8%, highlighting the limited spatial scope for the species’ long-term persistence.

The strong spatial correspondence between observed occurrences and model-predicted core habitats indicates that *Lepidium olgae* is largely restricted to a small number of environmentally stable microhabitats that function as centers of its realized niche. Accordingly, strengthening in situ conservation through strict protection of core populations and their habitats should be applied cautiously, only as a complementary measure.

Given the high environmental sensitivity of the species, integrating systematic field monitoring with periodic updates of SDM predictions is essential for supporting adaptive, evidence based management. Translocation should be applied cautiously and only as a complementary measure, guided by ensemble model outputs and limited to highly suitable yet currently unoccupied areas, while minimizing impacts on donor populations.

Overall, this study demonstrates how ensemble SDM results can be translated into biologically meaningful and spatially explicit conservation guidance, providing a robust scientific foundation for species specific conservation planning for *Lepidium olgae* and other narrow endemic mountain plants.

## Figures and Tables

**Table 1:** Area (km^2^) and percentage (%) of ensemble-predicted occurrence probability classes of *Lepidium olgae* within the study area.

Probability Class	Area (km^2^)	% of Reserve
<10%	233.29	61.2
10–50%	125.79	33.0
50–80%	12.20	3.2
>80%	9.91	2.6

**Table 2:** Spatial correspondence between observed occurrences and ensemble-predicted suitable habitats of *Lepidium olgae* within the study area.

Category	Area (km^2^)	% of Reserve	Explanation
Predicted suitable area	30.29	8.0	Area classified as suitable using TSS (Youden) threshold
Observed distribution	25.04	6.5	Area derived from field occurrence records
Overlap (observed + predicted)	24.69	6.48	Area where predicted suitability coincides with observations
Predicted only	5.81	1.32	Suitable area predicted by the model without observations
Observed only	0.56	0.09	Observed occurrences not predicted as suitable

## Data Availability

Environmental datasets used in this study are publicly available from the respective data providers. Due to conservation concerns regarding this endangered endemic species, precise occurrence coordinates are not publicly available but may be shared with qualified researchers upon reasonable request to the corresponding author.

## Supplementary Materials

The following supporting information can be downloaded at: https://www.mdpi.com/article/10.3390/plants15071125/s1, Table S1: List of bioclimatic variables used in the study.
