# Supplementary material for: Ecological Niche Modeling of the Narrow-Range Endangered Endemic Lepidium olgae in Uzbekistan
Source: Plants (Basel). 2026 Apr 7;15(7):1125. doi: 10.3390/plants15071125 (PMC13074910; doi:10.3390/plants15071125)
Supplement: Supplementary file 1 [file plants-15-01125-s001.zip › plants-4208976-supplementary.pdf]

Table S1. List of bioclimatic variables used in the study.

**Code**	**Variable Name**	**Description**
BIO1	Annual Mean Temperature	Average annual temperature
BIO2	Mean Diurnal Range	Mean of monthly (max temp – min temp)
BIO3	Isothermality	BIO2/BIO7 × 100
BIO4	Temperature Seasonality	Standard deviation ×100
BIO5	Max Temperature of Warmest Month	Maximum temperature of warmest month
BIO6	Min Temperature of Coldest Month	Minimum temperature of coldest month
BIO7	Temperature Annual Range	BIO5 – BIO6
BIO8	Mean Temperature of Wettest Quarter	Mean temperature of wettest quarter
BIO9	Mean Temperature of Driest Quarter	Mean temperature of driest quarter
BIO10	Mean Temperature of Warmest Quarter	Mean temperature of warmest quarter
BIO11	Mean Temperature of Coldest Quarter	Mean temperature of coldest quarter
BIO12	Annual Precipitation	Total annual precipitation
BIO13	Precipitation of Wettest Month	Monthly max precipitation
BIO14	Precipitation of Driest Month	Monthly min precipitation
BIO15	Precipitation Seasonality	Coefficient of variation
BIO16	Precipitation of Wettest Quarter	Total precipitation of wettest quarter
BIO17	Precipitation of Driest Quarter	Total precipitation of driest quarter
BIO18	Precipitation of Warmest Quarter	Total precipitation of warmest quarter
BIO19	Precipitation of Coldest Quarter	Total precipitation of coldest quarter
